# Advancements in Disease Modeling and Drug Discovery Using iPSC-Derived Hepatocyte-like Cells

**DOI:** 10.3390/genes13040573

**Published:** 2022-03-24

**Authors:** Josef Blaszkiewicz, Stephen A. Duncan

**Affiliations:** Department of Regenerative Medicine and Cell Biology, Medical University of South Carolina, Charleston, SC 29425, USA; blaszkie@musc.edu

**Keywords:** induced pluripotent stem cells, drug discovery, high-throughput screening, liver diseases

## Abstract

Serving as the metabolic hub of the human body, the liver is a vital organ that performs a variety of important physiological functions. Although known for its regenerative potential, it remains vulnerable to a variety of diseases. Despite decades of research, liver disease remains a leading cause of mortality in the United States with a multibillion-dollar-per-year economic burden. Prior research with model systems, such as primary hepatocytes and murine models, has provided many important discoveries. However, progress has been impaired by numerous obstacles associated with these models. In recent years, induced pluripotent stem cell (iPSC)-based systems have emerged as advantageous platforms for studying liver disease. Benefits, including preserved differentiation and physiological function, amenability to genetic manipulation via tools such as CRISPR/Cas9, and availability for high-throughput screening, make these systems increasingly attractive for both mechanistic studies of disease and the identification of novel therapeutics. Although limitations exist, recent studies have made progress in ameliorating these issues. In this review, we discuss recent advancements in iPSC-based models of liver disease, including improvements in model system construction as well as the use of high-throughput screens for genetic studies and drug discovery.

## 1. Introduction

Liver disease contributes to tens of thousands of deaths each year in the United States alone [1,2]. The multibillion-dollar economic burden associated with this mortality and required patient care has been growing in recent years and is projected to continue growing larger [3]. The term “liver disease” encompasses a wide variety of diseases with an equally large range of etiologies. There is a clear need for increased understanding of and effective treatments for these diseases. As such, many types of model systems have been developed and employed to serve this purpose.

Primary hepatocytes taken from living or recently deceased patients have classically been used for studying liver disease in vitro. As these cells are genetically identical to the donor, they allow for the identification and study of genes and mutations relevant to the patient’s specific pathology. However, primary hepatocytes rapidly de-differentiate when cultured, losing their native physiology, rendering long-term functional studies difficult [4]. Limited donor availability and invasive procedures associated with primary cell procurement also present significant obstacles, both for the study of rare diseases and for constructing high-throughput assays used for drug discovery and development.

Immortalized human liver cell lines provide a means of overcoming the insufficient supply of primary cells for large-scale studies. These cells can be maintained and expanded easily, making them amenable for high-throughput screening. However, these cells also show significantly altered metabolism associated with their malignant genotypes [5,6]. For example, the HepG2 hepatoma-derived cell line has severely defunct lipoprotein metabolism, which is an important physiological function of the liver that plays a role in many disease processes [7]. As such, these systems are not optimal for studying such metabolic processes or for discovering potential therapeutics that will target them.

Rodent models have been highly useful for both drug discovery and modeling human disease. In liver research, these systems provide an advantage over primary hepatocytes in terms of availability and amenability to genetic manipulation, especially with the rise of genomic editing tools, such as the Clustered Regularly Interspaced Short Palindromic Repeats (CRISPR)/Cas9 system [8]. They also allow for whole-animal studies to discover the impact of liver processes and novel drugs on the entire organism, which provide a significant advantage over monocultured cell lines. However, metabolic differences between rodents and humans present an obstacle for both drug discovery and the study of metabolic diseases [9,10]. In an effort to address these issues, avatar mouse models have been generated with functional human cells replacing the endogenous mouse hepatocytes [11,12]. Studies performed in such humanized animals can have higher relevance to human physiology than regular rodents [13,14]. The generation and maintenance of avatar mice remains an expensive and time-consuming process and so is more suited to safety and efficacy testing during drug development rather than as a platform for a primary, high-throughput drug screen.

Induced pluripotent stem cells (iPSCs) are a relatively new technology that can address the limitations of other model systems. iPSCs can be generated from a patient’s somatic cells via the induction of certain transcription factors—namely c-Myc, Klf4, Oct3/4, and Sox2 [15]. These reprogrammed cells have the ability to differentiate into endoderm-, mesoderm-, and ectoderm-derived tissue, including hepatocytes [15,16,17,18]. Importantly, they can be expanded and maintained practically indefinitely in culture, making them useful for large-scale assays. This property also makes them suitable for the study of rare diseases, as a single sample of somatic cells taken from a patient can give rise to a nearly limitless supply of iPSCs for further study. Unlike primary human hepatocytes, hepatocyte-like cells (HLCs) generated from iPSCs can be maintained without de-differentiating and losing physiological function, providing the opportunity for longitudinal studies without repeated invasive biopsies to obtain primary liver tissue [16,19]. Additionally, many physiological functions, such as lipoprotein metabolism, are maintained in HLCs, which provides a significant advantage over both immortalized cell lines and rodent models for studying these processes and the associated pathologies [7,20]. Thus, iPSC-based systems provide an attractive means for modeling liver disease and conducting high-throughput screens for drug discovery and development.

## 2. Differentiation of Hepatocyte-like Cells from iPSCs

iPSCs can be differentiated to hepatocyte-like cells over a 20-day period using recombinant growth factors and culture conditions that mimic those experienced by hepatocyte precursors in the developing human fetus [16,19]. Although there are many variations, in our own laboratory, pluripotent cells are first differentiated to endodermal tissue by exposure to activin A, fibroblast growth factor 2 (FGF2), and bone morphogenic protein 4 (BMP4) for 2 days, followed by activin A alone for 3 more days. Additional exposure to FGF2 and BMP4 under hypoxic conditions directs the endoderm to a defined hepatic fate, and 5 days of treatment with hepatocyte growth factor (HGF) produces immature hepatocytes resembling those found in a human fetus. Transfer to normoxic conditions and growth for 5 days in hepatocyte culture medium (HCM) supplemented with oncostatin M (OSM) leads to the development of more mature hepatocyte-like cells. This approach is both efficient and reproducible, allowing for sustained generation of large numbers of hepatocytes for use in high-throughput assays [16,19].

**Figure 1:**
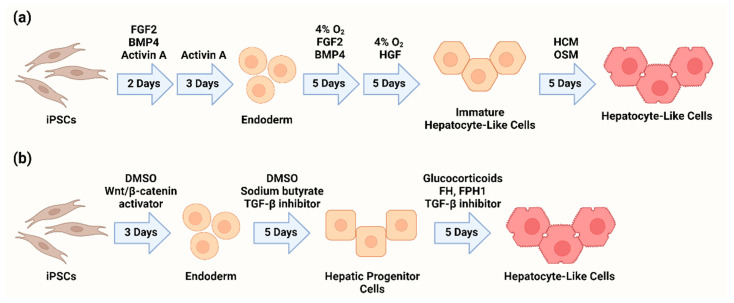
Abridged protocols for the differentiation of hepatocyte-like cells from iPSCs using (**a**) recombinant growth factors [13,16] or (**b**) small molecules [21].

As an alternative to the use of growth factors, small molecule regimens have been developed for the generation of HLCs from iPSCs. These regimens typically involve three phases, for example using Wnt/β-catenin activators to produce endoderm, TGF-β inhibitors to define the hepatic fate, and a small molecule/glucocorticoid mixture to drive hepatocyte maturation and proliferation [21]. The resulting hepatocytes show similar differentiation efficiency, physiological function, and transcriptomic profiles compared to growth factor-directed HLCs [21,22,23]. Small molecule-based protocols involve decreased time to maturation as well as lower costs compared to growth factor-based methods, presenting an attractive approach for the large-scale industrial production of HLCs. Figure 1 illustrates two protocols for generating hepatocyte-like cells from iPSCs.

## 3. Limitations and Recent Improvements in HLC Differentiation and Maturation

While iPSC-derived hepatocytes show numerous advantages as a model system for liver disease, they are not without their limitations. Chief among these is the well-documented difference in Phase I and II enzyme expression between iPSC-derived hepatocytes and mature primary hepatocytes [24,25,26,27,28,29]. These enzymes include members of the cytochrome P450 (CYP) family and are crucial for metabolizing and detoxifying compounds that pass through the liver. As such, they are of paramount importance in drug development, presenting an obstacle for the use of iPSC-based systems in drug toxicity studies. Recently, however, advancements in HLC generation have sought to improve the expression profile of these enzymes. Fluorescence-activated cell sorting can be used to isolate HLC subpopulations with more optimal transcriptional profiles by selecting for enriched mitochondrial content [30] or for surface markers, such as asialoglycoprotein receptor 1 (ASGR1) [31]. Small molecule compounds that improve the morphology and expression profile of HLCs have been identified and can be supplemented or substituted for certain growth factors in differentiation protocols [21,32,33]. Additionally, supplementing culture media with excess amino acids can improve HLC maturation and CYP expression [25]. These methods and others in development will ultimately contribute to iPSC-based systems that more faithfully replicate in vivo expression profiles of human hepatocytes.

In the liver, hepatocytes perform different physiological processes based on their three-dimensional arrangement in lobular units. These functional differences are largely regulated by cell–cell and cell–extracellular matrix (ECM) interactions based on the hepatocyte’s position in the lobule [34]. Monolayer cultures of iPSC-derived hepatocytes fail to completely recapitulate these interactions. Some approaches to overcome this include the use of plates coated with ECM substitutes, such as laminin, Matrigel, or recombinant E-cadherin (ECAD-Fc) [15,35,36]. Coculture methods also seek to improve upon this issue by providing other cell types for HLC interaction. In addition to improved HLC maturation [37], these approaches have the added benefit of permitting the study of disease processes that involve the interaction between hepatocytes and other cell types, such as sinusoidal epithelium and stellate cells in liver fibrosis [38].

Other techniques involve the construction of three-dimensional scaffold matrices that can then be populated with cells. HLCs grown on these scaffolds demonstrate higher expression of CYP family genes and decreased expression of fetal hepatocyte markers when compared with 2D-cultured HLCs, indicating a better maturation more suited for toxicology studies [39,40,41]. Three-dimensional organoids can also be generated using monoculture or coculture methods, resulting in improved physiological function without the need for engineered scaffolds. While previous organoid models suffered from lack of scalability, current production methods have been optimized for high-throughput applications [42]. For example, Ramli et al., 2020 developed organoids containing functional hepatocytes and cholangiocytes that recapitulated changes in structure and gene expression seen in patients with non-alcoholic fatty liver disease when exposed to high levels of fatty acids [43]. Organoids containing functional iPSC-derived cholangiocytes can model biliary diseases, such as primary sclerosing cholangitis [44] and cystic fibrosis-associated cholangiopathy [45]. Other organoid-based systems have been shown to be useful for interrogating genetic function [46] as well as for modeling inborn errors of metabolism [47].

**Figure 2:**
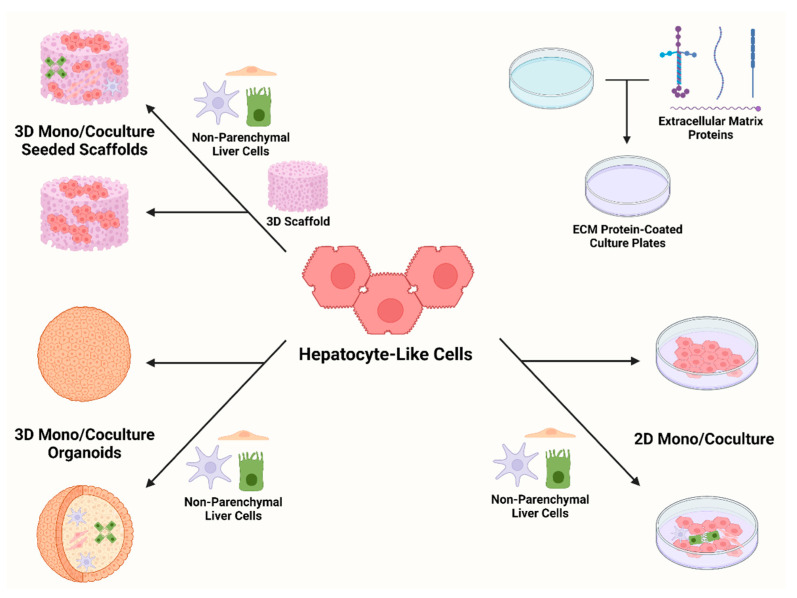
Methods of culturing iPSC-derived hepatocyte-like cells.

Advancements in HLC differentiation and modeling have also proven useful in studying infectious diseases such as malaria and hepatitis B. An iPSC-based model of malaria was improved by using a small molecule to induce CYP expression such that antimalarial drugs requiring CYP bioactivation could be effectively tested [48]. Coculture and organoid HLC models showed higher hepatitis B virus (HBV) infection efficiency than monocultured 2D methods, allowing for better propagation and study of the virus’ interaction with its host cells [49,50]. Providing more robust platforms for these pathogens that closely mimic their host environments in vivo is essential for understanding their pathophysiology and for seeking new treatments for the diseases they cause. Figure 2 summarizes current methods for culturing hepatocyte-like cells.

## 4. Genetic Function Studies

iPSC-derived HLCs can be used effectively for high-throughput studies of gene function. Genome-wide association studies (GWAS) have facilitated the identification of many genes potentially involved in a variety of pathologies. However, the correlative nature of these studies requires that further validation of identified candidates be performed. iPSC-based models are a viable approach for further exploring the contributions of allelic variations to liver disease. Abbey et al., 2020 used an iPSC-derived HLC organoid model to investigate the GWAS-identified *TRIB1* gene and found a lipid metabolism phenotype similar to that seen in a *TRIB1*-deficient mouse model, supporting a functional role for *TRIB1* in humans [46]. Tian et al., 2019 used iPSCs to confirm the functional roles of *GPC1* and *ADD3*, which had been associated with biliary atresia in a prior GWAS [51]. iPSC-based systems can also be used to establish cohorts for future GWAS studies, allowing identification and subsequent verification of functional variants seen in the cohorts [52,53]. Disease-specific, patient-derived iPSC panels have been established for α-1 antitrypsin (AAT) deficiency [54] and familial hypercholesterolemia (FH) [55], providing platforms for the discovery of functional variants related to these diseases and their severity.

The amenability of iPSCs to genetic manipulation via tools such as CRIPSR/Cas9 make these systems attractive for modeling and correcting liver diseases with genetic etiologies. Jing et al., 2018 created a model of mitochondrial DNA (mtDNA) depletion syndrome using CRISPR to knock out deoxyguanosine kinase in iPSCs, recapitulating the decreased mtDNA copy number and impairing the mitochondrial function seen in mtDNA depletion syndrome patients [56]. CRISPR-edited iPSCs also allowed for the exploration of a genetic variant associated with resistance to hepatitis B, specifically identifying the variant as a loss-of-function allele for a cellular receptor that mediates viral entry [57]. Genetic manipulation can also be used to correct mutations in patient-derived iPSCs, with the ultimate goal of generating disease-corrected hepatocytes for autologous transplantation back into the patients [58,59,60]. Improvements are also being made in gene-editing methodologies. For example, CRISPR/Cas9 co-targeting of the ATP1A1 channel confers digoxin resistance upon cells that receive desired genome editing, allowing for the highly efficient selection of clones with the desired edit [61].

## 5. Disease Modeling Using iPSC-Derived Hepatocyte-like Cells

As previously stated, iPSCs show great promise for modeling genetic disorders and can faithfully recapitulate pathologies seen in patients. Familial hypercholesterolemia is an autosomal dominant disorder associated with deficiency in the low-density lipoprotein receptor (LDLR). The absence or loss of function in this receptor leads to profoundly increased lipoprotein secretion by the liver in addition to impaired clearance of LDL from the blood. This dysregulation vastly increases blood lipoprotein levels, leading to premature and severe adverse cardiovascular effects [62]. Cayo et al. generated iPSCs from an extensively studied fibroblast cell line derived from an FH patient. Resulting hepatocyte-like cells showed impaired LDLR function, as well as greatly increased lipoprotein secretion, indicating that the model could be used to replicate FH pathology at a cellular level [63]. While this study dealt with an autosomal dominant disorder, recessive genetic disorders can be effectively modeled as well. Glycogen storage disease type II, also known as Pompe disease, is an autosomal recessive disorder that stems from the lack of lysosomal α-glucosidase. This results in the toxic accumulation of glycogen in the liver, heart, and skeletal muscle, with consequences that can be as severe as death in infancy [64]. iPSC-derived HLCs generated from affected patient cells showed enlarged lysosomes full of glycogen. Importantly, treating these HLCs with standard-of-care therapy using recombinant α-glucosidase ameliorated this accumulation, demonstrating the use of this model in exploring treatment options [65].

In addition to genetic disorders, environmental pathologies can also be modeled using iPSC-derived hepatocytes. Tian et al., 2016 exposed mature HLCs to pathological concentrations of alcohol and observed a dose-dependent increase in markers associated with mitochondrial DNA damage, lipid accumulation, and hepatocellular carcinoma. Strikingly, immature HLCs that more closely modeled human fetal hepatocytes did not show this increase in pathological markers although there was a significant reduction in proliferating cells [66]. Studies such as this could lead to a deeper understanding of the mechanism of hepatotoxic environmental agents and how their impact may differ between the fetal and postnatal stages of human development. Infectious diseases of the liver can also be successfully modeled using iPSCs. Although hepatoma lines are able to act as a host for infectious agents, their aberrant physiology can lead to the alteration of the pathogens’ biological processes as they adapt to their new host. HLCs, however, do not drive this adaptation and so can allow for a better understanding of the infectious agent’s biology [67]. Beyond providing an improved platform for the laboratory maintenance and propagation of liver pathogens, iPSC-derived HLCs have also proven valuable for studying host–pathogen interactions. For example, using HLCs as a host for the hepatitis C virus (HCV) allowed researchers to evaluate the activation of the innate immune response in infection, which was previously unattainable in hepatoma cell lines [68,69]. Table 1 provides a summary of recently developed iPSC-based models of liver disease.

## 6. High-Throughput Screens for Drug Discovery and Development

In addition to mechanistic studies of liver disease, iPSC-based models can be used for high-throughput assays to discover and develop novel therapeutics. Jing et al., 2018 conducted a small molecule screen using their model of mtDNA depletion syndrome, resulting in the identification of NAD+ as a potential treatment [56]. Cayo et al., 2017 identified cardiac glycosides as an effective means of reducing cholesterol secretion by the liver by conducting a large screening assay with their patient-derived model of familial hypercholesterolemia [13]. Both of these studies utilized a collection library consisting of over 2300 compounds, the majority of which are already approved for therapeutic use in humans. Conducting screens with such libraries provides the attractive benefit of identifying hits with prior approval for use in humans, which could expedite the process of repurposing the treatment for additional disorders [13,56]. Similar approaches have been taken to identify treatments for other diseases. For example, Parafati et al., 2020 used an HLC-based platform to screen 13,000 compounds for the ability to lower HLC intracellular lipid levels. Further exploration of the hits identified by this screen revealed a shared mechanism of action that could prove useful for treating non-alcoholic fatty liver disease [95]. HLCs can also be used to validate treatment candidates identified by a prior high-throughput screen. Völkner et al., 2022 used HLCs as a secondary screen to identify potential treatments for Niemann–Pick Disease Type C, an autosomal recessive disorder that leads to the accumulation of cholesterol and sphingolipids in the lysosome. In the study, candidates identified by a primary in-silico screen were further validated by their ability to rescue cholesterol accumulation in differentiated HLCs [86]. High-throughput assays using hepatocyte-like cells have also proven useful for identifying regulatory mechanisms of important processes, such as hepatocyte differentiation [96], as well as stellate cell activation and liver fibrosis [97].

The suboptimal expression of xenobiotic enzymes in iPSC-derived HLCs have historically hampered their use in toxicity studies. However, multiple groups have recently developed iPSC-based systems with improved expression profiles for use in toxicity screening. These systems possess unique designs and conditions, but each demonstrate results comparable to those obtained from studies with primary hepatocytes [98,99,100,101,102]. For example, Shinozawa et al., 2021 developed an efficient method of generating HLC organoids which show albumin secretion, cytochrome P450 activity, and expression of bile acid metabolism-related genes comparable to primary hepatocytes [100]. These systems provide scalable and highly physiologically relevant means to perform high-throughput toxicity assays without the functional and/or financial limits imposed by primary hepatocytes, hepatoma lines, and rodent models. Table 2 summarizes recent studies involving the use of iPSC-based models for high-throughput screening and toxicity assays.

## 7. Summary and Future Perspectives

iPSC-derived hepatocyte-like cells are an extremely useful technology for the study of liver disease. Although HLC-based models are not without their constraints, advancements are continuing to be made in improving their functional relevance and increasing their scalability. The number of diseases that can be successfully modeled with HLCs is continually expanding, opening the door for a deeper understanding of these diseases and the discovery of new treatments for them. Improvements in HLC-based toxicity studies raise the prospect of eventually tailoring drug therapy to individual patients based on data generated from cells sharing their exact genotypes. Additionally, the development of more disease-specific iPSC panels will allow for the identification of potential therapeutic targets and novel therapeutics for both common and rare liver diseases.

## Figures and Tables

**Table 1:** Liver Diseases Modeled by iPSC-based Systems.

Disease	Configuration	Model Characteristics	Reference
Abetalipoproteinemia	2D culture	Decreased ApoB secretion, intracellular lipid accumulation, increased cell death	[70]
Alagille Syndrome	3D organoids containing hepatocytes and cholangiocytes	Impaired bile duct formation and regenerative capacity	[71]
	2D culture	Patient-derived, HLC phenotype uncharacterized	[72]
Alcohol-induced Liver Injury	2D culture	Reduced proliferation, oxidative mitochondrial injury, increased steatosis, and hepatocellular carcinoma markers	[66]
α-1 Antitrypsin Deficiency	2D culture	AAT retention, enrichment of fibrosis- and cirrhosis-associated pathways	[54]
Autosomal Recessive Hypercholesterolemia	2D culture	Reduced LDL uptake	[73]
Bile Salt Export Pump (BSEP) Deficiency	2D culture	Impaired biliary excretion, altered localization of BSEP protein	[74,75]
Biliary Atresia	2D culture	Decreased biliary marker expression, increased expression of fibrosis markers	[51,76]
Citrullinemia Type I	3D organoids	Accumulation of ammonia, decreased ureagenesis	[47]
	2D culture	Decreased ureagenesis	[77]
Familial Hypercholesterolemia	2D culture	Inability to uptake LDL	[60]
Hemophilia B	3D organoids	Production of inactive coagulation factor IX (F9)	[78]
	2D culture	Reduced expression and activity of F9	[58]
	2D culture	Aberrant splicing of F9 mRNA leading to reduced F9 expression	[79]
Hepatitis B	3D organoids	Higher susceptibility to HBV infection than 2D culture, increased duration of infectious virus production	[49]
	2D coculture of HLCs and liver non-parenchymal cells	Improved efficiency of infection relative to 2D monoculture due to epidermal growth factor (EGF) modulation of endocytosis	[50]
Hepatitis C	2D culture	Permissive to infection with HCV, upregulation of type I and III interferons in response to infection	[68]
	2D culture	Higher susceptibility to and propagation of HCV compared to Huh7 cells	[80]
	2D culture	Supportive of full HCV life cycle, increased expression of interferon-stimulated genes	[69]
Hepatitis E	2D culture	Permissive host for hepatitis E virus natural isolates	[67]
Malaria	2D culture	Permissive host for infection with *Plasmodium* species; chemical maturation allows for bioactivation of primaquine	[48]
mtDNA Depletion Syndrome	2D culture	Decreased mtDNA copy number, disruption of mitochondrial ultrastructure, reduced mitochondrial respiration/intracellular ATP, increased reactive oxygen species levels	[56]
	2D culture and 3D organoids	Decreased mtDNA, reduced mitochondrial respiration, increased reactive oxygen species, increased sensitivity to iron overload	[81]
Nonalcoholic Fatty Liver Disease	2D culture	Patient-specific lipid droplet formation upon administration of oleic acid, decreased lipid metabolism-associated gene expression in higher levels of steatosis	[82]
	2D culture	Decreased electron transport chain activity, altered transcription of mitochondrial respiration pathways, increased pyruvate carboxylase activity, and fumarate accumulation in response to steatosis induction	[83]
	2D culture	Defects in V-ATPase assembly leading to increased ApoB secretion	[84]
	3D organoids with hepatocytes, macrophages, mesenchymal stem cells, and endothelial cells	Spontaneous lipid accumulation in absence of fatty acid supplementation	[85]
Niemann–Pick Disease Type C (NPC)	2D culture	Increased lysosomal accumulation of cholesterol, increased trafficking of NPC1 to lysosomes	[86]
	2D culture	Increased lysosomal cholesterol accumulation, increased cell size, upregulated autophagy and impaired autophagic flux	[87]
Ornithine Transcarbamylase Deficiency	2D culture	Decreased urea secretion	[88]
Pompe Disease	2D culture	Accumulation of glycogen in lysosomes	[65]
Primary Sclerosing Cholangitis	3D organoids containing cholangiocytes	Altered organoid morphology, increased cellular senescence and inflammatory cytokine secretion	[44]
Propionic Acidemia	2D culture	Knockout of propionyl CoA carboxylase	[89]
Transthyretin (TTR) Amyloidosis	2D culture	Secretion of abnormal TTR, increased expression of transferrin and unfolded protein response signaling pathways	[90,91]
Wilson Disease	2D culture	Patient-derived, HLC phenotype uncharacterized	[92]
	2D culture	Increased trafficking of ATP7B to the Golgi complex, increased rate of ATP7B degradation	[93]
Zellweger Spectrum Disorder	2D culture	Defective peroxisome assembly	[94]

**Table 2:** High-Throughput Drug Discovery and Development Assays Using iPSC-based Systems.

Disease/Purpose	Assay Description	Results	Reference
α-1 Antitrypsin Deficiency	Screened over 3000 compounds from the Johns Hopkins Drug library using immunofluorescence to determine effect on AAT levels	Five hits confirmed to cause consistent reduction in AAT across multiple iPSC lines	[103]
Liver Fibrosis	Screened over 1400 compounds using a red fluorescent protein reporter line to assay inhibition of stellate cell activation	Two compounds suitable for oral administration identified as potential treatments for liver fibrosis	[97]
Familial Hypercholesterolemia	Screened over 2300 small molecules from the SPECTRUM collection drug library using an ELISA-based assay to detect ApoB secretion	Identified cardiac glycosides as potential treatment for lowering ApoB secretion	[13]
mtDNA Depletion Syndrome	Screened over 2300 small molecules from the SPECTRUM collection drug library using a luciferase ATP assay to identify drugs that could restore ATP levels	Identified NAD as being able to improve ATP production and mitochondrial function	[56]
Niemann–Pick Disease Type C	Used a series of 2-hydroxypropyl-cyclodextrins to determine impact on cholesterol accumulation and hepatic function	Identified HPGCD as potential treatment for NPC	[87]
Non-alcoholic Fatty Liver Disease	Screened 13,000 compounds from AstraZeneca chemogenic library using BODIPY staining to quantify intracellular neutral lipid droplets	21 confirmed hits identified CDK2-4-C/EBPα/DGAT2 pathway as therapeutic target for lowering lipid accumulation	[95]
Identifying regulatory pathways for hepatic differentiation	Screened over 1100 small molecules using immunofluorescence to quantify HNF4α levels	Identification of HSP90β as a regulator of hepatic progenitor formation	[96]
Toxicity Screening	Generated mCherry-tagged CYP1A1 HLCs and screened 241 chemicals to identify aryl hydrocarbon receptor modulators	Five novel hits determined to up- or down-regulate expression of CYP1A1 in HLCs	[98]
	Developed a 3D coculture model with macrophages and endothelial cells; screened 159 known toxic compounds for effects on hepatic function	Identified albumin expression assay as most-sensitive method for calculating TC_50_ values with this system	[99]
	Screened 238 marketed drugs using liver organoids in a multiplexed readout assay	Validated high predictive values for effects on viability, cholestatic, and/or mitochondrial toxicity	[100]
	Developed noncontact coculture model of liver spheroids and renal proximal tubule cells to assay liver and kidney toxicity simultaneously	Demonstrated toxicity profiles could be discriminated with known toxic CYP inhibitor compound CsA	[101]
	Screened 47 compounds for effects on albumin, urea, and ATP levels using micropatterned coculture of HLCs and murine embryonic fibroblasts	Micropatterned coculture model showed similar sensitivity for toxic drug identification to primary hepatocyte model	[102]

## Data Availability

Not applicable.
